# Correction to: Pakistan Randomized and Observational Trial to Evaluate Coronavirus Treatment (PROTECT) of Hydroxychloroquine, Oseltamivir and Azithromycin to treat newly diagnosed patients with COVID-19 infection: A structured summary of a study protocol for a randomized controlled trial

**DOI:** 10.1186/s13063-022-06080-8

**Published:** 2022-02-15

**Authors:** Javed Akram, Shehnoor Azhar, Muhammad Shahzad, Waqas Latif, Khalid Saeed Khan

**Affiliations:** 1 Vice Chancellor/Professor of Internal Medicine, University of Health Sciences, Lahore, Pakistan; 2 Public Health, University of Health Sciences, Lahore, Pakistan; 3 Professor of Pharmacology, University of Health Sciences, Lahore, Pakistan; 4 Data Analyst at University of Health Sciences, Lahore, Pakistan; 5 Distinguished Investigator at Department of Preventive Medicine and Public Health, University of Granada, Granada, Spain

**Correction to: Trials 21, 702 (2020)**

**https://doi.org/10.1186/s13063-020-04616-4**

Following the publication of the original article [1], we were notified of the below corrections:

1- In the Intervention and Comparator section, Phosphate is erroneously mentioned.

2- In the Participants section, “Participants of any gender or age group having tested positive” should read “Participants of either gender above 18 years of age”.
